# Alkyl Polyglucosides as Components of Water Based Lubricants

**DOI:** 10.1007/s11743-012-1428-y

**Published:** 2013-01-05

**Authors:** Marian Włodzimierz Sułek, Marta Ogorzałek, Tomasz Wasilewski, Emilia Klimaszewska

**Affiliations:** Department of Chemistry, University of Technology and Humanities in Radom, Chrobrego 27, 26-600 Radom, Poland

**Keywords:** Friction coefficient, Wear, Lubricants, Surfactants

## Abstract

Water can be used as an ecological lubricant base if it is possible to select additives which can beneficially modify its tribological and corrosion properties. Additionally, those additives should not be harmful to human health and the natural environment. These conditions limit or even eliminate the possibility for the application of the additives used in traditional oil bases as they are insoluble in water and often toxic. Alkyl polyglucosides (APGs) have been suggested as additives improving lubricating properties of water. They are biodegradable and do not have to be recycled. They exhibit surface activity. They produce micelles at low concentration and lyotropic liquid crystals at high concentration. Two types of alkyl polyglucosides differing in alkyl chain lengths and degrees of polymerization were used in this investigation. Tribological tests were carried out using a ball-on-disc T-11 tester. The balls were made of steel, whereas the discs were made of steel, aluminium oxide, zirconium oxide, polyamide and poly(methyl methacrylate). The description of the device and the methods has been given in the literature (Szczerek and Tuszyński in TriboTest 8:273–284, 2002). The addition of APGs improves the lubricating properties of water. The relative decrease in motion resistance and wear depends both on the type of friction couple and on the kind of alkyl polyglucoside used. The tribological test results obtained were correlated with the activity of APGs measured as wettability of friction couples by their solutions.

## Introduction

There are a number of publications discussing water lubrication of friction couples made of ceramics [2, 3], plastics and composites [4, 5]. Friction couples made of steel were coated [6, 7] or corrosion inhibitors were added to water [8]. The analysis of the data from the literature [2–8] indicates that pure water does not satisfy the requirements that lubricating substances should meet. Hence, it is necessary to use appropriate additives which can reduce motion resistance and wear, counteract scuffing, inhibit the corrosive action of water and increase its viscosity. Surfactants seem to be the appropriate substances, primarily due to their ability to adsorb at the interface and to produce ordered structures in solutions [9–11].

New additives which have a beneficial effect on the lubricating and physicochemical properties of water as a lubricant have been described in our papers [12–16]. From among the compounds tested so far, special attention should be paid to alkyl polyglucosides (APGs), mainly due to the fact that they are biodegradable and do not have a harmful effect on humans and the natural environment.

## Alkyl Polyglucosides as Lubricity Modifiers for Water

**Fig. 1:**
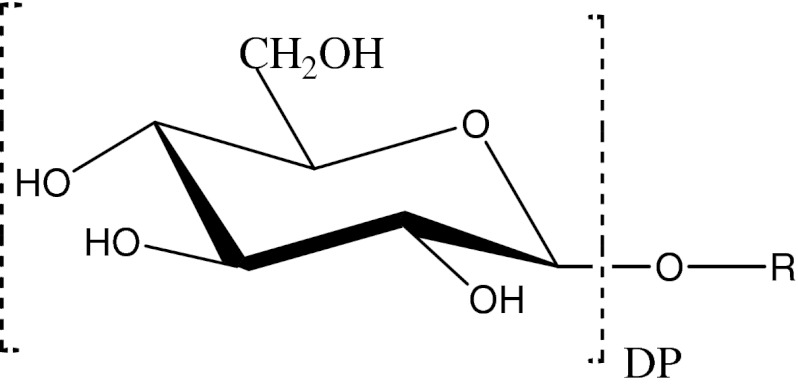
Molecular structure of alkyl polyglucoside (APG)

Alkyl polyglucosides (Fig. 1) are a group of surface active compounds.

Their hydrophilic and hydrophobic parts are clearly separated. The hydrophilic group in an APG molecule usually consists of one to five condensed glucoside parts (DP = 1–5). The hydrophobic group in APG molecules is an alkyl chain containing usually from six to eighteen carbon atoms. Surface activity depends both on the hydrophilic part and the hydrophobic part, although to varying degrees. Surface activity of surfactants is determined primarily by the chain length. According to Traube’s rule, surface activity at the water/air interface increases threefold with an increase in the chain length by a CH_2_ group (at 20 °C). The rule cannot be directly applied to the solid/solution interface but it gives an idea about the influence of the hydrophobic part. Therefore, shorter-chain alkyl polyglucosides (*n* < 6) exhibit low surface activity which limits their wide application.

The hydrophilic part affects adsorbability to a lesser extent. However, it affects solubility in polar solvents. Depending on the structure of the molecule, alkyl polyglucosides exhibit different solubility in various solvents. The compounds with the degree of polymerization (DP) from 1 to 5 containing alkyl chains whose length is from 6 to 14 carbons are soluble in water. The molecules with 16 to 22 carbon atoms are oil-soluble. In general, increasing the degree of polymerization increases solubility in a polar medium while lengthening of the alkyl chain increases solubility in a non-polar medium [17, 18]. From the viewpoint of the application of APGs as additives improving lubricating properties of water, these compounds must be both water-soluble and highly surface active. The adsorption of APGs on the surfaces of friction couples results in the formation of a surface structure whose composition will determine the formation of a lubricant film.

The physicochemical properties of alkyl polyglucosides are interesting. They occur in the form of monomers at low concentrations and produce micelles above the critical micelle concentration (CMC) usually of the order of hundredths of a percent. Aqueous solutions of alkyl polyglucosides may appear in the form of lyotropic liquid crystals at the concentrations of above 30 % [12, 15].

APGs used as additives considerably reduce motion resistance and wear and also improve antiseizure properties [12, 15]. So far, steel–steel friction couples have been the main research subject. It is thus interesting to analyze if APGs will have similarly beneficial tribological properties in the friction couples whose elements are made of various materials.

The aim of this research was to determine the effect of the kind of friction couple material on tribological properties of aqueous solutions of APGs. The experimental part presents the investigation on the effect of the concentration of aqueous solutions of two kinds of APGs on surface activity: surface tension (*σ*) and wetting angle (*θ*). This is followed by tribological tests on 1 % solutions. The tests are used to evaluate motion resistance and wear of friction couple elements.

## Experimental Procedures

### Materials

Two surfactants produced by Cognis and containing alkyl polyglucosides APG C_8_–C_10_ and APG C_12_–C_14_ were used in this investigation. Glucopon 600CSUP is a 50 % aqueous solution of alkyl polyglucosides with 12–14 carbon alkyl chains (APG C_12_–C_14_) and the average degree of polymerization DP = 1.4. Glucopon 215CSUP is a 63 % aqueous solution of alkyl polyglucosides with 8–10 carbon alkyl chains (APG C_8_–C_10_) and the average degree of polymerization DP = 1.5.

The tribological tests were carried out for water and 1 % aqueous solutions of two kinds of alkyl polyglucosides: APG C_8_–C_10_ and APG C_12_–C_14_. The friction couple elements were balls 6.35 mm in diameter made of 100 Cr6 and discs 25 mm in diameter and 8 mm in thickness made of steel, aluminium oxide (Al_2_O_3_), zirconium oxide (ZrO_2_), polyamide 6 (PA6) and poly(methyl methacrylate) (PMMA).

### Methods

Surface tension and wettability of the surfaces of the materials by solutions of the compounds containing additives with concentrations of 0.001, 0.005, 0.010, 0.055, 0.100, 0.500 and 1.000 % were a measure of surface activity of alkyl polyglucosides. Surface tension was determined by means of a Lauda tensiometer using the ring detachment method.

The wettability of various materials was determined using a drop shape analysis technique. A Krüss instrument was used for the tests which were carried out at 22 °C. The wetting angles of metal (steel), ceramic (Al_2_O_3_ and ZrO_2_) and polymer (PA6 and PMMA) surfaces were measured. All the surfaces were prepared using the same procedure as the one used for the tribological tests.

A T-11 tribological tester produced by ITeE Radom (Poland) was used. The measurements were carried out according to the methods described in the literature [1]. All the elements of the friction couples were chemically cleaned before the tests. The time of the test run was 900 s, the load of the friction couple was 50 N and the sliding speed was 0.1 m/s. Based on friction force measurements the coefficient of friction was calculated from the formula:

$$ \mu = \frac{{F_{T} }}{P} $$

where *F*_*T*_ friction force [N], *P* pressure [N].

Friction coefficient values are given in Figs. 4 and 5. Figure 4 analyzes the dependence of the coefficient of friction on time, while Fig. 5 shows averaged values of *μ* from the whole 900 s test and three independent measurement series. The measure of error is a standard deviation of the arithmetic mean at an assumed confidence level of 0.95. Wear scar profiles of the balls and discs were analyzed after the tribological tests. The device used for the investigation was a Profilometer TOPO produced by the Institute of Advanced Manufacturing Technology in Kraków (Poland).

## Results and Discussion

### Surface Activity of Aqueous Solutions of Alkyl Polyglucosides

Surface activity forms the basis for the application of alkyl polyglucosides (APGs) as additives modifying lubricating properties of water. Therefore, the presentation of the investigation results is preceded by a discussion on the effect of APGs on their affinity for the surface.

The problem of contact of a lubricant with a solid is essential from the tribological point of view. The surface phase and the bulk phase can be distinguished at the interface in a solution. A “blurred” boundary can be found between the two phases. Due to adsorption from the solutions, the surface phase is enriched with the component which shows a higher affinity for the surface. It is characteristic that individual components compete for “free sites” on the surface.

The problems of adsorption at the interface become more complex when solutions contain surfactants which can form micelles both in the surface phase and in the bulk phase. Surfactants occur in solutions in the form of monomers in a low concentration range but after exceeding the critical surface aggregation concentration (CSAC) they produce surface micelles [9–11, 18]. The formation of micelles in the surface phase terminates at a concentration corresponding to the critical micelle concentration (CMC) in the bulk phase. A considerable decrease in surface tension (*σ*) and wetting angle (*θ*) of APG solutions relative to water is a confirmation of high surface activity of alkyl polyglucosides.

**Fig. 2:**
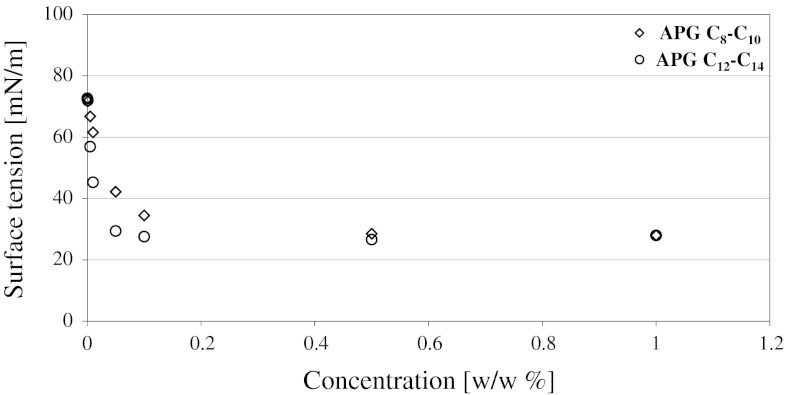
Dependence of surface tension on concentration of aqueous APG solutions

Changes in the *σ* value as a function of APG concentration in water are characteristic of surfactant solutions (Fig. 2).

At low concentrations, of the order of hundredths of a percent, a sudden drop occurs in the value of *σ* followed by its stabilization at higher concentrations. As expected, at lower concentrations (*c* < 0.1 %) long-chain APGs (APG C_12_–C_14_) have a lower *σ* value and, thus, higher surface activity than short-chain APGs (APG C_8_–C_10_). This results from the hydrophobic effect whose influence increases with an increase in the hydrophobic part—in this case, an increase in the alkyl chain length. The hydrophobic effect has also an influence on the CMC value which is lower for the APG C_12_–C_14_ solutions [13, 17–18]. In the case of higher concentrations (*c* > 0.1 %) the *σ* values are comparable for both compounds which indicates formation of the surface phase. A thermodynamic equilibrium between monomers and micelles in the surface phase and the bulk phase is reached. APGs in 1 % solutions have equal values of surface tension which are about 2.5-times lower than those for water.

**Fig. 3:**
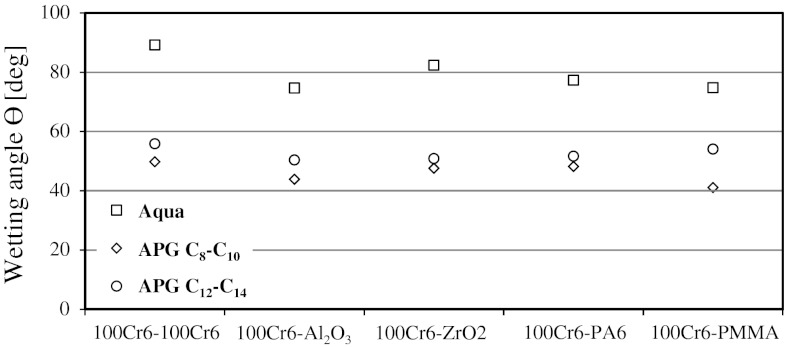
Values of wetting angles *θ* for various types of materials

The results of the investigation of wettability of various materials by 1 % aqueous solutions of APG C_8_–C_10_ and APG C_12_–C_14_ are shown in Fig. 3.

Good wettability of the surfaces of the materials tested can be observed even at APG concentrations of the order of tenths of a percent. A particularly sharp decrease in the wettability angle occurs at low concentrations (*c* < 0.1 %) while at higher concentrations (*c* > 0.2 %) there are no significant changes in the measured value. Particular attention was paid to the 1 % solutions used as lubricating substances in the tribological tests (Sect. 4.2). Contrary to expectations, APG C_8_–C_10_ solutions show better wettability of the materials tested than APG C_12_–C_14_ solutions. One might expect higher wettability by long-chain alkyl polyglucosides which exhibit higher affinity for surfaces. This may indicate that the entropy connected with the formation of surface structures has a decisive effect on the free enthalpy of the adsorption reaction. It is difficult to determine the gradation of changes in the wetting angle for individual materials as the differences in the measured values of the wetting angle (*θ*) are relatively small. The angle *θ* for APG C_12_–C_14_ solutions ranges from 50° to 56° while for APG C_8_–C_10_ solutions it ranges from 41° to 50°. The measuring error can be estimated as ±2°. As a result, the interpretation of changes for the 1 % solutions poses a considerable risk. The analysis of wettability of the materials by aqueous APG solutions leads to the conclusion that 1 % APG solutions show significantly better wettability of the surfaces of the materials tested than water and the wetting angle is from 1.7 to 1.8 times higher for APG C_8_–C_10_ solutions and from 1.5 to 1.6 times higher for APG C_12_–C_14_ solutions than the angle for water. The results given clearly indicate that the effect of the type of material (steel, polymer or ceramics) is not very significant. It can thus be stated that wettability of steel, ceramic and polymer surfaces by 1 % APG solutions is comparable.

### Tribological Characteristics of Aqueous Alkyl Polyglucoside Solutions in Various Friction Couples

Tribological properties of aqueous solutions of alkyl polyglucosides (APGs) were characterized by motion resistance and wear.

**Fig. 4:**
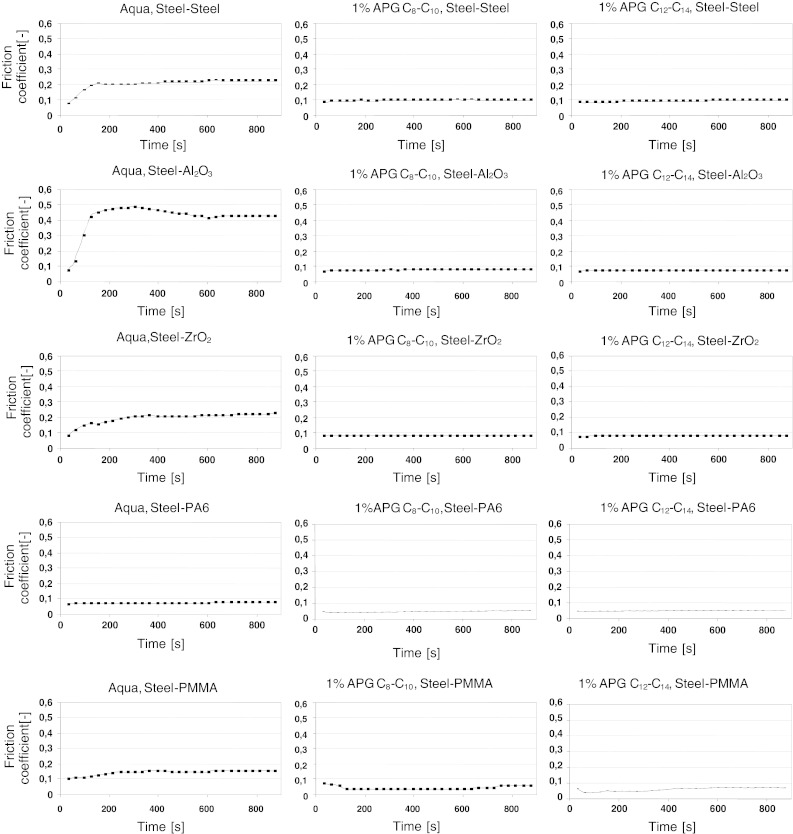
Dependencies of friction coefficient on time for water and 1 % aqueous solutions of APGs for various friction couples

The dependences of the coefficient of friction on water and aqueous APG solutions are shown in Fig. 4.

In the presence of water, the coefficient of friction (*μ*) increases within the first 100 s of the test and then stabilizes at a certain level or tends to increase slightly. The highest increase in the value of *μ* can be observed for the discs made of Al_2_O_3_ and, successively, for discs made of steel, ZrO_2_, PMMA and PA6. It can be assumed that the increase for the steel-polyamide 6 couple is negligibly small. The dependencies change when aqueous APG solutions are the lubricating substances. Practically from the first seconds of the friction process, the *μ* values remain constant, and for the steel-polymer couple, particularly for the discs made of PMMA, the coefficients of friction decrease slightly.

**Fig. 5:**
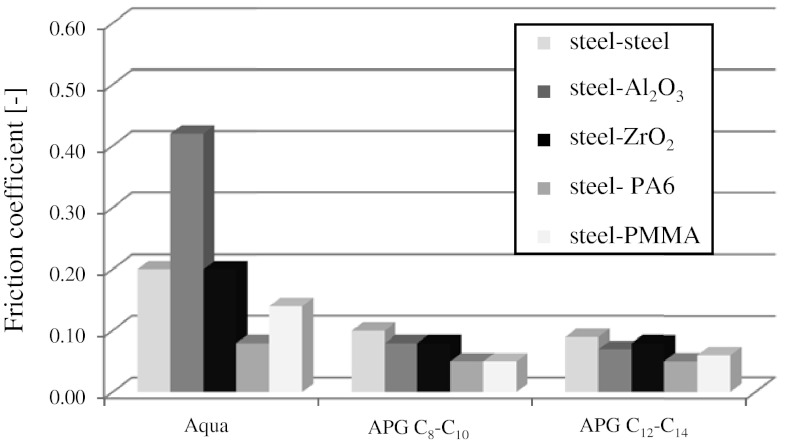
Dependencies of averaged friction coefficient on type of friction couple materials for water and 1 % aqueous solutions of APG

The mean *μ* values for individual couples are given in Fig. 5.

Practically no effect of the kind of alkyl polyglucosides on motion resistance can be observed. However, in the case of all the friction couples tested, there is a pronounced drop in the *μ* value after adding any of the alkyl polyglucosides to water. The friction coefficient values decreased 2-, 5-, 2-, 1.5- and 2.5-fold for the steel–steel, steel-Al_2_O_3_, steel-ZrO_2_, steel- PA6 and steel-PMMA couples, respectively. The smallest changes can be observed for the steel-PA6 couple where the *μ* value equals 0.08 in the presence of water and 0.05 in the presence of the solutions. Depending on the kind of disc material, the following gradation of the friction coefficient value can be given: *μ* (PA6) ≤ *μ* (PMMA) ≤ *μ* (Al_2_O_3_) ≤ *μ* (ZrO_2_) < *μ* (steel).

**Fig. 6:**
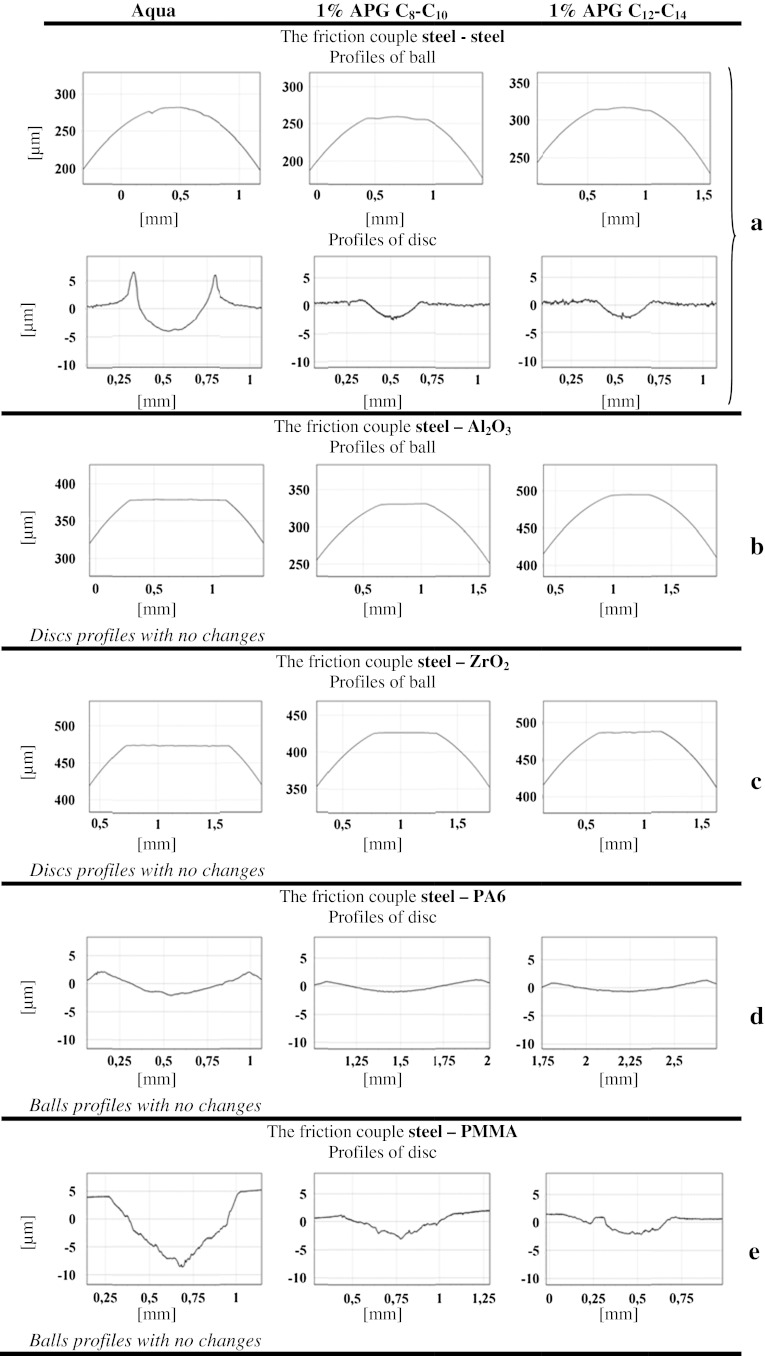
Profiles of ball and disc scars after frictional tests

Due to various mechanisms of wearing of the materials tested, it is difficult to estimate and compare wear in terms of absolute values. Changes in the measured ball and disc profiles were adopted as a criterion for wear measure. Figure 6 shows the most characteristic profiles for individual friction couples and the lubricating substances used.

Figure 6 shows only those friction couple elements whose profile has changed under friction. On the basis of the results obtained, it is possible to notice the effect of individual materials. In the case of the steel–steel (100Cr6-100Cr6) couple in water it is mainly the disc which undergoes wear with a characteristic displacement of material to outside the friction area. In the presence of alkyl polyglucoside solutions both the ball and the disc undergo wear and its intensity is considerably lower (Fig. 6a). When the discs are made of ceramics, only the steel ball undergoes wear and the analysis of the ball’s profile indicates its frictional wear (Fig. 6b, c). In the case of the discs made of plastics (PA6 and PMMA) only the discs undergo wear which is connected with plastic deformations (Fig. 6d, e). The addition of APGs to water results in a decrease in wear for all the friction couples.

## Conclusions

Alkyl polyglucosides (APGs) are chemical compounds which are used in numerous applications in various branches of industry. They are biodegradable and safe for humans and the natural environment. APGs used as additives reduce motion resistance and wear relative to water. The effect of the additives on a decrease in the coefficient of friction (*μ*) is particularly important. It decreases even several times relative to water used as a base. The differences between short-chain (APG C_8_–C_10_) and long-chain (APG C_12_–C_14_) alkyl polyglucosides are small. As expected, the quantity and type of wear depend on the materials of friction couples. Wear is lower in the presence of APG solutions than in the presence of water.

The results obtained will serve as a basis for formulating multicomponent aqueous solutions as metalworking and fire-resistant hydraulic fluids. In these types of operational fluids, alkylpolyglucosides will act as lubricity additives having a high ecological value.
